# Characteristics of Elderly Long-Term Care Residents Who Were Injured and Transferred to Hospital Emergency Departments in Korea: A Retrospective Multicenter Study

**DOI:** 10.1155/2019/7803184

**Published:** 2019-06-02

**Authors:** Myeong Namgung, Keon Kim, Dong Hoon Lee, Ho Young Yune, Jung Hee Wee, Duk Ho Kim, Eui Chung Kim, Jee Yong Lim

**Affiliations:** ^1^Department of Emergency Medicine, Chung-Ang University Hospital, Seoul 06973, Republic of Korea; ^2^Department of Emergency Medicine, Ewha Womans University Hospital, Seoul 07985, Republic of Korea; ^3^Department of Emergency Medicine, College of Medicine, Chung-Ang University, Seoul 06973, Republic of Korea; ^4^Department of Emergency Medicine, Hallym University, Dongtan Sacred Heart Hospital, Hwaseong-si, Gyeonggi-do 18450, Republic of Korea; ^5^Department of Emergency Medicine, College of Medicine, Yeouido St. Mary's Hospital, The Catholic University of Korea, Seoul 07345, Republic of Korea; ^6^Department of Emergency Medicine, Eulji University, Seoul 01830, Republic of Korea; ^7^Department of Emergency Medicine, CHA University School of Medicine, Seongnam-si 13496, Republic of Korea; ^8^Department of Emergency Medicine, Seoul St. Mary's Hospital, Seoul 065691, Republic of Korea

## Abstract

The objective of this retrospective multicenter study was to investigate the mechanism and characteristics of trauma experienced by patients aged ≥65 years who were transferred from a long-term care hospital to one of five university hospital emergency departments. Of 255,543 patients seen in one of the five emergency departments, 79 were transferred from a long-term care hospital because of trauma. The most common trauma mechanism was slipping down, with 33 (58.9%) patients, followed by falling from a bed (17.9%), striking an object such as a wall or corner (10.7%), overextending a joint (8.9%), and unknown mechanisms (3.6%). Many cases of slip (39.4%) occurred in relation to the bathroom. Comparing slip and fall from a bed, we found more hip fractures (95.2%) because of slipping down than falling from a bed (57.1%); traumatic brain injury only occurred in slip cases. These traumas cause significant morbidity in elderly patients; therefore, we sought to identify strategies that prevent slip in long-term care hospitals.

## 1. Introduction

With the improvement of medicine and economy, life expectancy has increased and the elderly population is increasing rapidly. According to the Statistics Korea database, the number of people aged ≥65 years has increased from 10.2% in 2008 to 14.3% in 2017 and is expected to reach 41.0% by 2060 [1]. As the size of the elderly population increases, so will medical demand by elderly patients with chronic disease. In addition, the number of long-term care hospitals (LTCH) has increased steadily from 714 in 2009 to 1516 in 2017 [2].

Aging results in cognitive impairment, muscle weakness, loss of balance, impairment of vision and hearing, reduced response time for accident avoidance, and increased occurrence of underlying disease [3].Elderly patients in LTCHs have many comorbidities such as dementia, stroke, hypertension, diabetes, and poor mobility [4]. Therefore, the risk of trauma and mortality for elderly patients may be relatively high. Previous studies of elderly people experiencing falls showed that long-term care institutions have higher fall rates with more serious complications [5]. Therefore, as the number of patients in LTCHs increases, the number of patients transferred from LTCHs who experienced trauma will also increase.

However, there are few studies of the mechanism and characteristics of trauma experienced by elderly patients transferred from LTCHs in Korea. The majority of studies have focused on fall-induced trauma that was experienced by geriatric patients in LTCHs, mainly by falling from a bed and slipping down [6–8]. Therefore, this study aimed to investigate the mechanism and characteristics of trauma experienced by patients aged over 65 years who were transferred from an LTCH.

## 2. Materials and Methods

### 2.1. Study Setting and Participants

This multicenter, retrospective cohort study investigated all emergency department (ED) visits by LTCH patients aged ≥65 years between January 2017 and December 2017 among five university hospitals in the metropolitan area of Korea.

### 2.2. Data Sources

The data of patients who visited the ED between January 2017 and December 2017 were extracted from the National Emergency Department Information System (NEDIS) of Korea, a nationwide government system in operation since 2003 that collects data from more than 150 Korean emergency centers.

The following NEDIS data variables from patient ED visits were analyzed: patient demographic information (age, sex), triage acuity, chief complaint (nonmedical cause), initial mental status, primary diagnosis, and disposition. The Korean Triage System (KTAS) categorizes patients by a severity scale that ranges from 1 to 5, with 1 being the most severe.

After screening the NEDIS database, we reviewed the patients' electronic medical records (EMR) to assess for comorbidities, ambulation status, detailed mechanism of injury, final diagnosis, and Injury Severity Score (ISS). Ambulation status was divided into three categories based on EMR records: bedridden; ambulation with assistance, such as cane, walker, or other help; and ambulation without assistance. In a detailed review of injury mechanism, slip was defined as any fall occurring during ambulation except for falling from a bed. Our goal was to analyze the mechanism and the rate of trauma experienced by elderly patients in LTCHs.

### 2.3. Statistical Analysis

All statistical analyses were conducted using IBM SPSS Statistics for Windows version 23.0 (IBM Corp., Armonk, NY, USA). We entered descriptive statistics for the numbers and percentages of patients. The chi-squared test was used to compare slip and fall from a bed. Statistical significance was a P value less than 0.05.

### 2.4. Ethics Statement

This study was approved by the institutional review board of each hospital, and the need for patient informed consent was waived.

## 3. Results

**Figure 1:**
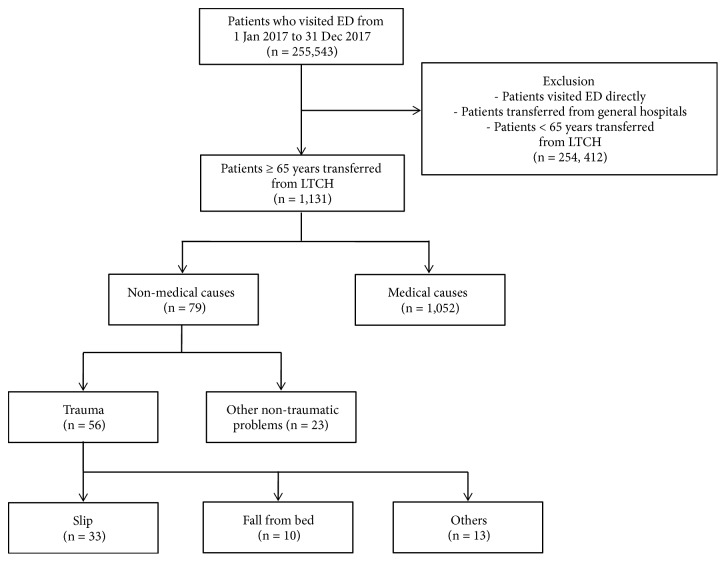
The flow chart of enrolled patients.

A total of 255,543 patients presented to one of five university hospital EDs in 2017. Of these, 1131 patients were transferred from an LTCH, and 79 were for nonmedical reasons. In the patients with nonmedical causes, 56 were trauma patients and 23 were nontraumatic injury patients (Figure 1).

Patient characteristics are shown in Table 1. The patients between the ages of 75 and 84 was the largest group of patients, 33 (41.8%). The most common underlying diseases were neurodegenerative, such as dementia and Parkinson's disease (17.1%), followed by hypertension (15.6%) and cerebrovascular disease (12.8%). Among all patients, 57.0% needed help in ambulation by use of a cane, walker, or assistance from another person, 25.3% were bedridden, and 17.7% were able to walk without help. The chief complaint during ED visits were pain in hip and pelvis (39.2%), followed by head pain (26.6%) and medical equipment problems (20.3%) such as catheter or tubing. In KTAS, grade 4 was the most common (48.1%), followed by grade 5 (22.8%) and grade 3 (21.5%). Patient care data showed that 38 (48.1%) returned to an LTCH with no abnormal findings, 31 (39.2%) were admitted to the general ward, and five (6.3%) were admitted to the ICU. Three (3.8%) of the patients who refused treatment were discharged. One patient died (1.3%) and one patient (1.3%) was transferred to another hospital because of emergent treatment needs (Table 1).

Fifty-six elderly trauma patients were transferred from an LTCH. The most common mechanism of trauma was slip, with 33 (58.9%) patients. Among the 33 patients who were injured by slip, 13 (39.4%) slipped in the bathroom or while coming in or out of the bathroom. Slip events most frequently resulted in hip fracture (60.6%), followed by cerebral concussion (18.2%) and traumatic brain injury (TBI) (12.1%). One patient may experience several types of injury, resulting in a sum of diagnoses exceeding 100%. Additional mechanisms of trauma were falling from a bed (17.9%), hitting against a wall or corner (10.7%), overextending a joint (8.9%), and unknown mechanisms (3.6%) (Table 2).

Among elderly patients transferred from an LTCH with nonmedical causes, 23 patients were transferred because of nontraumatic causes. The detailed histories of these patients were categorized by medical device related problems such as Foley catheter, drainage tube, or arteriovenous fistula (17 patients); presence of a foreign body in the gastrointestinal tract or upper airway (six patients) (Table 3).

Slip and fall from a bed accounted for the largest proportion of trauma mechanism; Table 4 presents the differences between these two groups. The need for ambulation assistance was the factor that separated the two groups. Patients who need ambulation assistance were the largest group with 69.7% in the slip group and 50.0% in the fall from a bed group. However, the next largest proportion of patients was those in the slip group who walk without assistance (27.3%) and bedridden patients in the fall from a bed group (40.0%). A slip event experienced by a bedridden patient was defined as falling while using a wheelchair during transfer. Other variables (age, sex, KTAS, mental status, ISS, and disposition) were not statistically different. Hip fracture was more common in the slip group (95.2%) than the fall from a bed group (57.1%), while TBIs only occurred in the slip group (Table 4).

## 4. Discussion

The objective of this study was to examine the detailed mechanism and characteristics of traumas experienced by elderly patients who were transferred from LTCHs. The mechanisms of trauma were categorized into four groups: slip, fall from a bed, hitting against something, and overextending movements. Previous studies included slip and fall from a bed in the ‘fall' category. However, this study distinguished between slip and fall from a bed events to confirm the differences between trauma mechanisms and identify preventable causes.

This study demonstrates that slip (61.1%) was more frequent than fall from a bed (18.5%) among elderly trauma patients transferred from LTCHs to the ED. These results were different from the results of a patient safety report about falls at medical institutions issued by the Korean Minister of Health and Welfare in March 2018. The patient safety report is a system to prevent accident recurrence by analyzing cases that are self-reported by medical institutions to identify risk factors and preventative measures. According to the patient safety report, the most common mechanism related to falls reported by medical institutions was fall from a bed (68.2%), and most falls (54.3%) occurred in the patient ward. However, as shown in Table 2 of this study, slip was the most common mechanism of trauma, and among slip events, 39.4% occurred in the bathroom or near the bathroom, but not in the ward.

A comparison of trauma mechanisms revealed that slip events, more often than fall from a bed events, are related to the ambulation assistance method in LTCHs. Table 4 shows that patients who need ambulation assistance were the most common in both groups, while patients who did not need ambulation assistance were the second most common in the slip group, and patients in a bedridden state were the second most common in fall from a bed group. This result was consistent with previous studies showing that the incidence of fall injuries among ambulatory patients was greater than that of nonambulatory patients [9, 10]. In other words, patients who were able to walk without assistance were likely to experience more slip events than patients who have difficulty walking without assistance, especially in an area of high risk for slipping such as a bathroom.

This study also demonstrates that hip fractures occur more often with slip events than with fall from bed events and that TBIs only occurred in association with slip events. Hip fracture and TBI had serious negative effects on the patients. Previous studies show the short-term mortality of elderly patients following hip fracture had a three- to sixfold increase during the first year [11–13]. The mortality rate from TBI among elderly people was two times higher than that among young people[14]. These high mortalities were associated with comorbidities such as neurological and kidney-related diseases and postoperative complications such as infection and heart failure [15, 16]. Traumas caused by slip events had high mortalities and poor outcomes among elderly patients. Therefore, it was important to prevent slip of elderly patients in LTCHs. In particular, as most cases of hip fracture occurred in a bathroom, strategies to prevent slip in bathrooms are necessary.

When the data of elderly patients with injury were extracted from the NEDIS, they were classified by medical and nonmedical causes. The nontraumatic mechanisms of nonmedical problems are presented in Table 3. Most problems (73.9%) were related to medical devices and six patients had foreign body presence as a diagnosis, which were common nonmedical problems in the LTCHs of Korea.

This study has several limitations. First, a major limitation was its retrospective design. Furthermore, the patient information and mechanism of trauma were insufficient because we could not confirm information at the LTCH where the injury occurred. Second, because this study used a multicenter design in a metropolitan area of South Korea, selection bias may have occurred which could have affected the generalizability of the results. Third, there may be selection bias in patients who decided to transfer to university hospitals. Because we only analyzed patients who were transferred from LTCHs, minor injuries that did not need evaluation and major injuries that were not transferred due to the family declining care were not included in this study. For these reasons, our results should be interpreted cautiously.

## 5. Conclusion

This study showed that the most common trauma mechanism of elderly patients transferred from LTCHs was slip and most frequently occurred in relation to a bathroom. More serious trauma such as hip fracture and TBI could occur in slip. Therefore, it is necessary to prevent slip in LTCHs.

## Figures and Tables

**Table 1:** General characteristics of patients aged ≥ 65 years with nonmedical cause transferred from long-term care hospital.

	N (%)
Age	
65-74 years	19 (24.1)
75-84 years	33 (41.8)
≥85 years	27 (34.2)
Sex	
Male	32 (40.5)
Female	47 (59.5)
Comorbidities	
Neurodegenerative disease	36 (17.1)
Hypertension	33 (15.6)
Cerebrovascular disease	27 (12.8)
Orthopedic disease	25 (11.9)
Diabetes	23 (10.9)
Cardiovascular disease	21 (10.0)
Renal disease	10 (4.7)
Psychiatric disease	8 (3.8)
Malignancy	5 (2.4)
Pulmonary disease	4 (1.9)
Liver disease	3 (1.4)
Others	16 (7.6)
Ambulation state	
Bed ridden	20 (25.3)
Ambulation with assist (cane, walker or other help)	45 (57.0)
Ambulation without assist	14 (17.7)
Chief complain	
Pain, hip and pelvis	31 (39.2)
Pain, head	21 (26.6)
Intervention (catheter or tube related)	16 (20.3)
Foreign body	4 (5.1)
Dyspnea	3 (3.8)
Oral bleeding	2 (2.5)
Pain, upper extremities	2 (2.5)
KTAS	
1	1 (1.3)
2	5 (6.3)
3	17 (21.5)
4	38 (48.1)
5	18 (22.8)
Disposition	
Admission	
General ward	31 (39.2)
ICU	5 (6.3)
Return to LTCH	38 (48.1)
Against discharge	3 (3.8)
Transfer to other hospital	1 (1.3)
Death	1 (1.3)

KTAS: Korean triage acuity scale, ICU: intensive care unit, LTCH: long-term care hospital.

**Table 2:** Detailed mechanism of trauma from long-term care hospital.

Mechanism	diagnosis	N (%)
Slip (n = 33)		
Slip in bathroom	Hip fracture	7 (21.2)
	Cerebral concussion	2 (6.1)
	Shoulder dislocation	1 (3.0)
	Contusion of hip	2 (6.1)
Slip on the way to bathroom	Hip fracture	2 (6.1)
	Cerebral concussion	1 (3.0)
	Sprain of wrist	1 (3.0)
Slip while walking	Traumatic SDH	3 (9.1)
	Hip fracture	2 (6.1)
	Contusion of face	2 (6.1)
	Cerebral concussion	1 (3.0)
Slip during rehabilitation therapy	Hip fracture	2 (6.1)
	Face laceration	1 (3.0)
	Cerebral concussion	1 (3.0)
Slip with wheelchair	Traumatic SDH	1 (3.0)
Unknown detailed mechanism	Hip fracture	7 (21.2)
	Cerebral concussion	1 (3.0)
	Corneal laceration	1 (3.0)
Fall from bed (n = 10)	Cerebral concussion	3 (30.0)
	Facial bone fracture	2 (20.0)
	Hip fracture	4 (40.0)
	Contusion of shoulder	1 (10.0)
	Contusion of hip	1 (10.0)
	Rib fracture	1 (10.0)
Hit against something (n = 6)	Cerebral concussion	4 (66.7)
	Hip fracture	2 (33.3)
	Contusion of face	1 (16.7)
Over-extended movement (n = 5)		
Excessive pull of patient's extremities by caregivers	Hip fracture	2 (40.0)
	Hip dislocation	1 (20.0)
	Humerus fracture	1 (20.0)
Open mouth too wide	TMJ dislocation	1 (20.0)

SDH: subdural hemorrhage, TMJ: temporomandibular joint.

**Table 3:** Detailed histories of patients who transferred from long-term care hospital because of other nontraumatic problems.

Category	Diagnosis	N (%)
Medical device related (n = 17)		
Foley catheter	Foley catheter occlusion	4 (23.5)
	Foley self-removal	1 (5.9)
PTBD and PTGBD	PTBD and PTGBD removal	4 (23.5)
Perm catheter and AVF	Perm catheter removal	2 (11.8)
	AVF occlusion	2 (11.8)
PEG tube	PEG tube malfunction	2 (11.8)
C-line	For C-line insertion	1 (5.9)
Tracheal tube	Tracheal tube removal	1 (5.9)
Foreign body (n = 5)	F.B. in gastrointestinal tract	2 (40.0)
	F.B. in upper airway	3 (60.0)
Others (n = 3)	Tongue laceration	1 (33.3)
	Hip dislocation	1 (33.3)
	Asphyxia	1 (33.3)

PTBD: percutaneous transhepatic bile drainage, PTGBD: percutaneous transhepatic gallbladder drainage, AVF: arteriovenous fistula, PEG: percutaneous endoscopic gastrostomy.

**Table 4:** Comparison of injury mechanism between slip and fall from a bed from long-term care hospital.

	Fall from bed (n = 10)	Slip (n = 33)	*p*-value
Age (years)	82.70 ± 9.30	80.12 ± 7.59	0.377
Sex, n (%)			0.153
Male	2 (20.0)	16 (48.5)	
Female	8 (80.0)	17 (51.5)	
Comorbidities, n (%)			
Neurodegenerative disease	4 (40.0)	16 (48.5)	
Hypertension	5 (50.0)	18 (54.5)	
Cerebrovascular disease	5 (50.0)	11 (33.3)	
Orthopedic disease	6 (60.0	7 (21.2)	
Diabetes	3 (30.0)	12 (36.4)	
Cardiovascular disease	0 (0.0)	7 (20.0)	
Renal disease	1 (10.0)	5 (15.2)	
Psychiatric disease	3 (30.0)	3 (9.1)	
Malignancy	0 (0.0)	1 (3.0)	
Pulmonary disease	0 (0.0)	2 (6.1)	
Liver disease	0 (0.0)	3 (9.1)	
Others	1 (10.0)	4 (12.1)	
Ambulation state, n (%)			0.005
Bed ridden	4 (40.0)	1 (3.0)	
Ambulation with assist	5 (50.0)	23 (69.7)	
Ambulation without assist	1 (10.0)	9 (27.3)	
KTAS, n (%)			0.125
1	0 (0.0)	0 (0.0)	
2	2 (20.0)	1 (3.0)	
3	4 (40.0)	10 (30.3)	
4	3 (30.0)	21 (63.6)	
5	1 (10.0)	1 (3.0)	
Mental status at ED visit, n (%)			0.572
Alert	9 (90.0)	31 (93.9)	
Verbal response	1 (10.0)	1 (3.0)	
Painful response	0 (0.0)	1 (3.0)	
No response	0 (0.0)	0 (0.0)	
Diagnosis, n (%)			
Fracture	7 (70.0)	21 (63.6)	
Head and face	2 (28.6)	1 (4.8)	
Hip and pelvis	4 (57.1)	20 (95.2)	
Ribs	1 (14.3)	0 (0.0)	
Contusion and dislocation	5 (50.0)	10 (30.3)	
Head and face	3 (60.0)	7 (70.0)	
Extremity	2 (40.0)	3 (30.0)	
Traumatic brain injury	0 (0.0)	4 (12.1)	
Others	0 (0.0)	3 (9.1)	
ISS	9.60 ± 3.10	11.00 ± 5.87	0.648
Disposition, n (%)			0.946
Admission	7 (70.0)	21 (63.6)	
General ward	6	17	
ICU	1	4	
Return to LTCH	3 (30.0)	10 (30.0)	
Against discharge	0 (0.0)	1 (3.0)	
Transfer to other hospital	0 (0.0)	1 (3.0)	

KTAS: Korean triage and acuity scale, ED: emergency department, ISS: injury severity score, ICU: intensive care unit, LTCH: long-term care hospital.

## Data Availability

The data used to support the findings of this study are available from the corresponding author upon request.
